# Transcriptional Profiling of Human Dendritic Cell Populations and Models - Unique Profiles of In Vitro Dendritic Cells and Implications on Functionality and Applicability

**DOI:** 10.1371/journal.pone.0052875

**Published:** 2013-01-14

**Authors:** Kristina Lundberg, Ann-Sofie Albrekt, Inge Nelissen, Saskia Santegoets, Tanja D. de Gruijl, Sue Gibbs, Malin Lindstedt

**Affiliations:** 1 Department of Immunotechnology, Lund University, Lund, Sweden; 2 Environmental Risk and Health Unit, Flemish Institute for Technological Research (VITO), Mol, Belgium; 3 Department of Medical Oncology, VU University Medical Center, Amsterdam, The Netherlands; 4 Department of Dermatology, VU University Medical Center, Amsterdam, The Netherlands; University of Bergen, Norway

## Abstract

**Background:**

Dendritic cells (DCs) comprise heterogeneous populations of cells, which act as central orchestrators of the immune response. Applicability of primary DCs is restricted due to their scarcity and therefore DC models are commonly employed in DC-based immunotherapy strategies and *in vitro* tests assessing DC function. However, the interrelationship between the individual *in vitro* DC models and their relative resemblance to specific primary DC populations remain elusive.

**Objective:**

To describe and assess functionality and applicability of the available *in vitro* DC models by using a genome-wide transcriptional approach.

**Methods:**

Transcriptional profiling was performed with four commonly used *in vitro* DC models (MUTZ-3-DCs, monocyte-derived DCs, CD34-derived DCs and Langerhans cells (LCs)) and nine primary DC populations (dermal DCs, LCs, blood and tonsillar CD123^+^, CD1c^+^ and CD141^+^ DCs, and blood CD16^+^ DCs).

**Results:**

Principal Component Analysis showed that transcriptional profiles of each *in vitro* DC model most closely resembled CD1c^+^ and CD141^+^ tonsillar myeloid DCs (mDCs) among primary DC populations. Thus, additional differentiation factors may be required to generate model DCs that more closely resemble other primary DC populations. Also, no model DC stood out in terms of primary DC resemblance. Nevertheless, hierarchical clustering showed clusters of differentially expressed genes among individual DC models as well as primary DC populations. Furthermore, model DCs were shown to differentially express immunologically relevant transcripts and transcriptional signatures identified for each model DC included several immune-associated transcripts.

**Conclusion:**

The unique transcriptional profiles of *in vitro* DC models suggest distinct functionality in immune applications. The presented results will aid in the selection of an appropriate DC model for *in vitro* assays and assist development of DC-based immunotherapy.

## Introduction

Dendritic cells (DCs) orchestrate immune responses by initiating and regulating T-cell responses. Immense efforts are being made to fully understand their physiology, as well as to develop DC-based immunotherapy [1] and predictive test systems [2]. However, the use of primary DCs is limited by their scarcity (<1% in peripheral blood) so to circumvent this, DCs derived *in vitro* are commonly employed. Model DCs can be differentiated from various precursors, such as the CD34^+^ cells in bone marrow, umbilical cord blood or peripheral blood, as well as CD14^+^ monocytes [3]–[7]. Although much has been gained with the development of *in vitro* DC models from primary precursors, these models are restricted by the heterogeneity derived from donor-to-donor variability and the requirement for donor material. Being a myeloid cell line, MUTZ-3 DCs do not suffer from these limitations [8], [9] and have proven valuable as cell basis in test assays predicting sensitization [10], [11] as well as for cancer vaccine development [12].

Several DC models are widely used to understand the physiology of primary DCs. However, the interrelationship between distinct DC models is not clarified and neither is their relative resemblance to specific primary DC populations. The latter task is complicated by the complexity of the *in vivo* DC network, where several subtypes with unique phenotypic and transcriptional profiles have been identified in different organs. By far the most abundant populations in blood and tonsils are the CD1c^+^ myeloid DCs (mDCs) and the CD123^+^ plasmacytoid DCs (pDC), however, other populations, such as the CD16^+^ DCs in blood and the CD141^+^ DCs in blood and tonsils, have also been identified [13], [14]. In skin, two main subtypes have been described, i.e. the Langerin/CD207^+^ epidermal Langerhans cells (LC) and the DC-SIGN/CD209^+^ dermal DCs (DDC) [15]. Transcriptional studies of DC subsets have proven valuable in understanding DC subset relationships. For example, Robbins et al. performed transcriptional analyses of primary DC subsets from mice and humans and suggested human CD141^+^ DCs to be counterparts of mouse CD8^+^ DCs, [16]. Also, Haniffa et al. used a transcriptional approach to demonstrate that CD141^+^ DCs isolated from skin are closely related to their counterparts in blood and homologous to mouse CD103^+^ or CD8^+^ DCs [17]. Regarding *in vitro* DC models, Robbins et al. showed that MoDCs were more closely related to *in vitro* derived macrophages than to primary blood DCs; however, no primary DCs isolated from tissues were included in that analysis and neither were other DC models. Thus, resemblance of *in vitro* DC models to each other and to primary tissue-DC subsets remains unclear.

Development of cell-based *in vitro* test systems for prediction of allergenicity of chemicals is urgently required to limit animal testing. The 7th Amendment to the Cosmetics Directive bans testing of cosmetic ingredients on animals in EU from 2013, yet the REACH (Registration, Evaluation and Authorization of Chemicals) legislation require that all chemicals are assessed for hazardous effects [18]. The central role of DCs in immune regulation, and in the sensitization process in particular [19], supports their use in predicting allergenicity *in vitro*. However, large cell numbers will be required and thus, test assays will be based on model DCs that mimic the detrimental response by primary DCs to sensitizing agents. To evaluate the applicability of individual DC models in allergenicity test assays, comparisons of *in vitro* DC models as well as understanding their resemblance to primary DC populations is warranted.

*In vitro* DCs are attractive tools in order to redirect detrimental or inadequate immune responses, and adoptive transfer of model DCs is being explored in treatment of e.g. cancer, autoimmunity and infectious diseases (reviewed in [20]–[23]). In immunotherapeutic applications, the suitability of specific model DCs depends on their ability to acquire desired attributes upon in vitro modulation. For example, induction of stable stimulatory or suppressive model DCs is of utmost importance as this determines whether immunity or tolerance is induced [24], [25]. Also, the ability to internalize antigen is vital in order to induce antigen-specific adaptive responses. Although the potential of DC-based immunotherapy has been recognized and explored for several years, clinical efficacy is still limited [20], [26]. However, identifying characteristics of specific DC models may direct further developments, leading to improved efficacy.

In the current study, global transcriptional analysis was used in order to understand functionality and applicability of *in vitro* DCs. Profiling of monocyte-derived DCs (MoDCs), CD34^+^-derived LCs (CD34-LCs), CD34^+^-derived DCs (CD34-DCs) and MUTZ-3 DCs gained insight into their interrelationship, as well as their resemblance to an array of primary DC populations. Furthermore, 155 immune-related genes expressed by *in vitro* DCs were identified and expression levels were compared to *ex vivo* DC populations. Finally, transcriptional signatures were identified for individual DC models, indicating functional diversity. The presented data give insight into DC physiology and the suitability of distinct DC models in test assays, as well as in immunotherapy.

## Methods

### Ethics Statement

Written informed consent was obtained from blood donors and from parents of underage donors of tonsils. The data was analyzed anonymously. Regarding cord blood donation, written informed consent was given by the mothers and the study was approved by the ethical commission of the University of Antwerp, Belgium. Human skin specimens were obtained from healthy donors undergoing corrective breast or abdominal plastic surgery after verbal informed consent according to the Dutch Code for secondary use of anonymous rest material.

### Isolation of DC subsets from human peripheral blood, tonsils and skin

Isolation of blood and tonsillar DC subsets has been described previously [14]. Briefly, tonsils, obtained from children undergoing tonsillectomy, were minced and incubated with 2 mg/ml collagenase IV and 100 U/ml DNase I for 15 min at room temperature. Mononuclear cells were isolated from the tonsillar single-cell suspensions or leukocyte-enriched buffy coats by Ficoll-Paque (Amersham Biosciences, Uppsala, Sweden) density gradient centrifugation. B cells, T cells and monocytes were depleted and negatively selected cells enriched for DCs were incubated with FITC-conjugated mAbs against CD3 (BD Bioscience, San Jose, CA), CD14 and CD19 (DakoCytomation A/S, Glostrup, Denmark), APC-conjugated anti-HLA-DR (BD Bioscience) and either PE-conjugated mAb against CD141 (Miltenyi Biotech, Bergisch Gladbach, Germany) or CD123 (BD Pharmingen, San Diego, CA). The DC-enriched cells from peripheral blood were also labeled with PE-conjugated CD16 mAb (BD Bioscience). CD1c^+^ DCs were incubated with a lineage specific PE-labeled antibody cocktail (DakoCytomation A/S), HLA-DR-APC (BD Bioscience) and CD1c-FITC (Miltenyi Biotech). Gating strategy for sorting of specific DC subsets from blood and tonsils is described in detail in Lindstedt et al. 2005 [14]. Briefly, upon gating of live cells in forward and side scatter, lineage negative and HLA-DR^high^ DCs, positive for either CD1c, CD141, CD123 or CD16 were gated and sorted on a FACSDiVa or a FACSAria (BD Bioscience). Dermal dendritic cells and Langerhans cells were isolated from human skin specimens obtained from healthy donors undergoing corrective breast or abdominal plastic surgery, as previously described [27]. Briefly, split thickness slices of skin containing both the epidermis and the dermis were isolated using a dermatome, cut in pieces (0.5 cm^2^) and incubated with 2.4 U/ml Dispase II (Roche Diagnostics, Mannheim, Germany) for 30–60 minutes at 37°C. The epidermis and dermis were separated with tweezers and washed. To isolate LC, the epidermal sheets were incubated with PBS containing 0,05% (v/v) trypsin (Invitrogen Life Technologies, Carlsbad, CA) for 10 minutes at 37°C, and the epidermal single-cell suspension was enriched for LC by density centrifugation over Lymphoprep (Nycomed AS, Oslo, Norway) and CD1a-guided magnetic cell sorting (MACS; Miltenyi Biotec, Bergisch Gladbach, Germany). To isolate dermal DC, the dermis was incubated with PBS containing 0.48 U/ml Dispase and 6 mg/ml Collagenase A (Boehringer Mannheim, Mannheim, Germany) at 37°C for 2 hours, after which single cell suspension was enriched for dermal DC by CD1a-guided magnetic cell sorting (MACS).

### Generation of *in vitro*-derived DCs

MoDCs were differentiated from peripheral blood monocytes (purified using CD14 positive selection by MACS) by culturing cells in rhIL-4 (50 ng/ml) and rhGM-CSF (150 ng/ml) for 7 days, as described previously [28]. CD34^+^ progenitor-derived dendritic (CD34-DC) and Langerhans-like cells (CD34-LC) were differentiated from cord blood. CD34^+^-cell isolation and culture procedures have been described before [29]. Briefly, human cord blood samples were collected from the umbilical blood vessels of placentas of normal, full-term infants. Mononuclear cells were separated from the cord blood by density gradient centrifugation and subsequently CD34^+^ progenitor cells were extracted by positive immunomagnetic selection (MACS). These cells were cultured for 12 days in Iscove's modified Dulbecco's medium (IMDM) containing 10% (v/v) fetal bovine serum, 500 ng/ml rhGM-CSF (Gentaur, Brussels, Belgium), 50 ng/ml rhSCF (Biosource, Nivelles, Belgium), 2.5 ng/ml rhTNF-α (Roche, Basel, Switzerland), and 34 ng/ml rhIL-4 (Biosource) to induce proliferation and differentiation towards immature CD34-DC, according to the method described by Lardon et al. [30]. To obtain Langerhans-like cells (CD34-LC), 5 ng/ml rhTGF-β (Gentaur) was additionally added to the medium from the first day of culture.

The human myeloid leukemia-derived cell line MUTZ-3 (DSMZ, Braunschweig, Germany) was maintained in α-MEM (Invitrogen, Paisley, UK), supplemented with 20% (v/v) fetal bovine serum (Hyclone Laboratories, Logan, UT) and 40 ng/ml rhGM-CSF (Leukomax) (Novartis, Basel, Switzerland), as described [31]. To generate dendritoid cells, MUTZ-3 cells (1×10^5^ cells/ml) were differentiated in the presence of rhGM-CSF (150 ng/ml) and rhIL-4 (50 ng/ml). Medium was exchanged every 2–3 days. After 7 days, cells were incubated with anti-CD1a-FITC (DakoCytomation A/S) and HLA-DR-PE (BD Bioscience) and live cells, gated based on forward and side scatter properties, were subsequently gated for HLA-DR and CD1a positivity (figure S1b) and FACSAria sorted to generate highly pure CD1a^+^ cells, referred to as MUTZ-3 DCs.

### Preparation of cRNA and gene chip hybridization

RNA isolation and gene chip hybridization was performed as previously described [14]. Briefly, cell pellets of freshly isolated primary DCs, *in vitro* differentiated MoDCs, CD34-DCs and CD34–LCs, from 3 different donors, as well as *in vitro*-derived MUTZ-3 DCs in triplicates, were dissolved in TRIzol Reagent (Life Technologies) and stored at −20°C. After chloroform extraction, total RNA was precipitated in isopropanol, rinsed with 70% ethanol, lyophilized, and dissolved in 10 µl of distilled water. Fragmentation, hybridization, and scanning of the Human Genome U133 Plus 2.0 Arrays were performed according to the manufacturer's protocol (Affymetrix, Santa Clara, CA). The preparation of labeled cRNA was performed according to the Two-cycle Eukaryotic Target Labeling assay protocol, using the GeneChip Expression 3′ amplification two-cycle labelling and control reagents kit (Affymetrix). Briefly, cDNA was generated from total RNA (>10 ng for all samples, in accordance with the GeneChip® Expression Analysis Technical Manual), using SuperScript II (Life Technologies, Carlsbad, California) and a T7-oligo(dT) promoter primer (Affymetrix). After a second-strand cDNA synthesis, cDNA was converted to cRNA by an *in vitro* transcription reaction (Life Technologies). Thereafter, the cRNA was purified using RNeasy Mini kit (Qiagen, Hilden, Germany), and the yield was controlled with a spectrophotometer. A second cycle of cDNA synthesis was performed, followed by the same cleanup as above and a second *in vitro* transcription reaction cycle with biotin-labelled ribonucleotides and T7 RNA polymerase. Labelled cRNA was purified, using RNeasy Mini kit (Qiagen), quality controlled with Agilent 2100 Bioanalyzer (Agilent Technologies, Santa Clara, CA), and denatured at 94°C before hybridization of 10 µg of the purified material. The samples were hybridized to Human Genome U133 Plus 2.0 Arrays at 45°C for 16 h by rotation (60 rpm) in an oven. The arrays were then washed, stained with streptavidin-PE (Invitrogen Molecular Probes), washed again, and scanned with a GeneArray Scanner (Affymetrix).

### Microarray data analysis

All cell types included in the transcriptional study and abbreviations used can be viewed in Table 1. Fluorescence intensity was analyzed using the GeneChip Operating Software (GCOS) 1.1 (Affymetrix), and scaled to a target value of 100. Data was uploaded into Expression Console 1.1 (Affymetrix), normalized with the MAS5 algorithm and for graphics, log transformed data (Robust Multi-Array (RMA)) was used. To ensure that other cell types were not contaminating the samples, mRNA encoding typical markers of T-cells, B-cells and NK-cells (CD3D, CD3E, CD3G, CD3z, CD8a, CD8b, TCRα, CD19 and CD56) were confirmed to have intensities <200, considered to be below positive expression (MAS5 normalized data) (data not shown). Background noise in the dataset was eliminated by a 20^th^ percentile cutoff in intensity signals, resulting in lists of 51,191 genes. (Microarray data generated from model DCs has been uploaded to ArrayExpress, accession number E-MEXP-3787.) Transcripts differentially expressed among all samples, as well as among *in vitro* DC models and *ex vivo* primary DC populations separately, were identified in Qlucore Omics Explorer 2.0 (Qlucore AB, Lund, Sweden) using ANOVA analysis with a p-value cutoff at <10^−6^ (corresponding to p<0.05 after Bonferroni correction). Furthermore, transcripts shared by the latter two groups were identified. Relationships among samples and populations were analyzed using principal component analysis (PCA). Briefly, PCA transforms a large set of parameters (transcripts in this case) into three summary variables (main components) which are illustrated as three axes (described in more detail in [32]). PCA is commonly used to analyze multidimensional data (reviewed in [33]) as it creates a 3D image that can instantly be interpreted in terms of interrelationship among samples. Similarities of replicate samples were demonstrated by the *k*-Nearest Neighbors (*k*-NN) algorithm (*2*-NN in this case) which is the Euclidean distance in gene space between samples. Furthermore, relationships between cell populations were demonstrated by the minimal spanning tree analysis (lines connecting the different populations). Additionally, hierarchical clustering was performed using Cluster 3.0, based on complete linkage and Euclidean distance measure [34], and heatmaps were subsequently produced using Java Treeview 1.0.12 [35]. In this manner, heatmaps were created to visualize profiles based on transcripts differentially expressed among all samples, among *in vitro* DC samples, and among *ex vivo* DC samples, respectively. Also, a dendogram of *in vitro* DC samples was produced in JMP (SAS Institute Inc., Cary, NC, USA) by hierarchical clustering of the differentially expressed transcripts using complete linkage.

**Table 1:** Human cell types included in the transcriptional study and abbreviations used.

Abbreviation	Cell types compared
	***In vitro models***
MUTZ3-DC	MUTZ3-derived dendritoid cells (CD1a^+^, differentiated with GM-CSF and IL-4)
MoDC	Peripheral blood monocyte-derived DC (CD1a^+^/CD14^−^, differentiated with GM-CSF and IL-4)
CD34-DC	Cord blood CD34^+^ progenitor cell-derived DC (differentiated with IL-4, TNF-α , GM-CSF and SCF)
CD34-LC	Cord blood CD34^+^ progenitor cell-derived Langerhans cells (differentiated with IL-4, TNF-α, GM-CSF, SCF and TGF-β)
	***Ex vivo populations***
tPDC	Tonsillar plasmacytoid DC (Lin^−^/HLA-DR^+^/CD123^+^)
bPDC	Blood plasmacytoid DC (Lin^−^/HLA-DR^+^/CD123^+^)
tCD1c-DC	Tonsillar CD1c^+^ DC (Lin^−^/HLA-DR^+^/BDCA-1^+^)
bCD1c-DC	Blood CD1c^+^ DC (Lin^−^/HLA-DR^+^/BDCA-1^+^)
tCD141-DC	Tonsillar CD141^+^ DC (Lin^−^/HLA-DR^+^/BDCA-3^+^)
bCD141-DC	Blood CD141^+^ DC (Lin^−^/HLA-DR^+^/BDCA-3^+^)
bCD16-DCs	Blood CD16^+^ DC (Lin^−^/HLA-DR^+^/CD16^+^)
DDC	Dermal DC (CD1a^+^/DC-SIGN^+^)
LC	Epidermal Langerhans cells (CD1a^+^/Langerin^+^)

To understand immunological features of *in vitro* DC models, gene expression was analysed to identify transcripts encoding TLRs, receptors, chemokines, lectins, TNF molecules, interleukins and CD antigens (based on information in Ingenuity pathway analysis (IPA, Ingenuity Systems, Mountain View, CA, USA) and NetAffx (Affymetrix)). Transcripts expressed by one or more *in vitro* DC models (MAS5 normalized data, average intensity >200) were selected and intensity levels graded on a four step scale based on average expression intensity. Corresponding expression levels by *ex vivo* blood, tonsil and skin DCs were additionally extracted from the microarray data and assigned similarly. Also, PCA analysis was performed based on the identified immune-related transcripts.

In order to pinpoint specific characteristics of the individual DC models, transcriptional signatures were identified. To this end, transcripts were selected based on three criteria: 1) Intensity level >2 fold higher as compared to each of the other *in vitro* DC models (calculated as the ratio of mean signal intensity of MAS5 normalized data) 2) Statistical significance (p<0.05, student's t-test) in each comparison 3) Mean intensity level >200. CD34-DCs and CD34-LCs were additionally compared together versus MoDCs and MUTZ-3 DCs.

## Results and Discussion

### *In vitro* and *ex vivo* DC phenotypes

Differentiated MUTZ-3 DCs, MoDCs, CD34-DCs and CD34-LCs showed appropriate and immature phenotypes with expression of CD1a and HLA-DR, whereas CD86 expressions were low (figure S1, S2 and S3, respectively). Furthermore, CD34-LCs and CD34-DCs were shown to lack CD83 expression and in contrast to CD34-DCs, CD34-LCs expressed CD207/Langerin (figure S2). Phenotypes of primary DCs have been published previously [14], [27]. In brief, tonsillar and blood DC subsets were shown to have immature phenotypes based on their lack of CD80 and low expression of CD86 [14]. Furthermore, LCs have been demonstrated to express CD207/Langerin and to be immature with low CD83 and CD86 expressions [27]. Although DDCs showed a similar low level of CD86, these cells were shown to express higher levels of CD83, before as well as after FACS sorting, and thus may be of a more activated phenotype.

### Relationship of *in vitro* and *ex vivo* DCs

**Figure 1:**
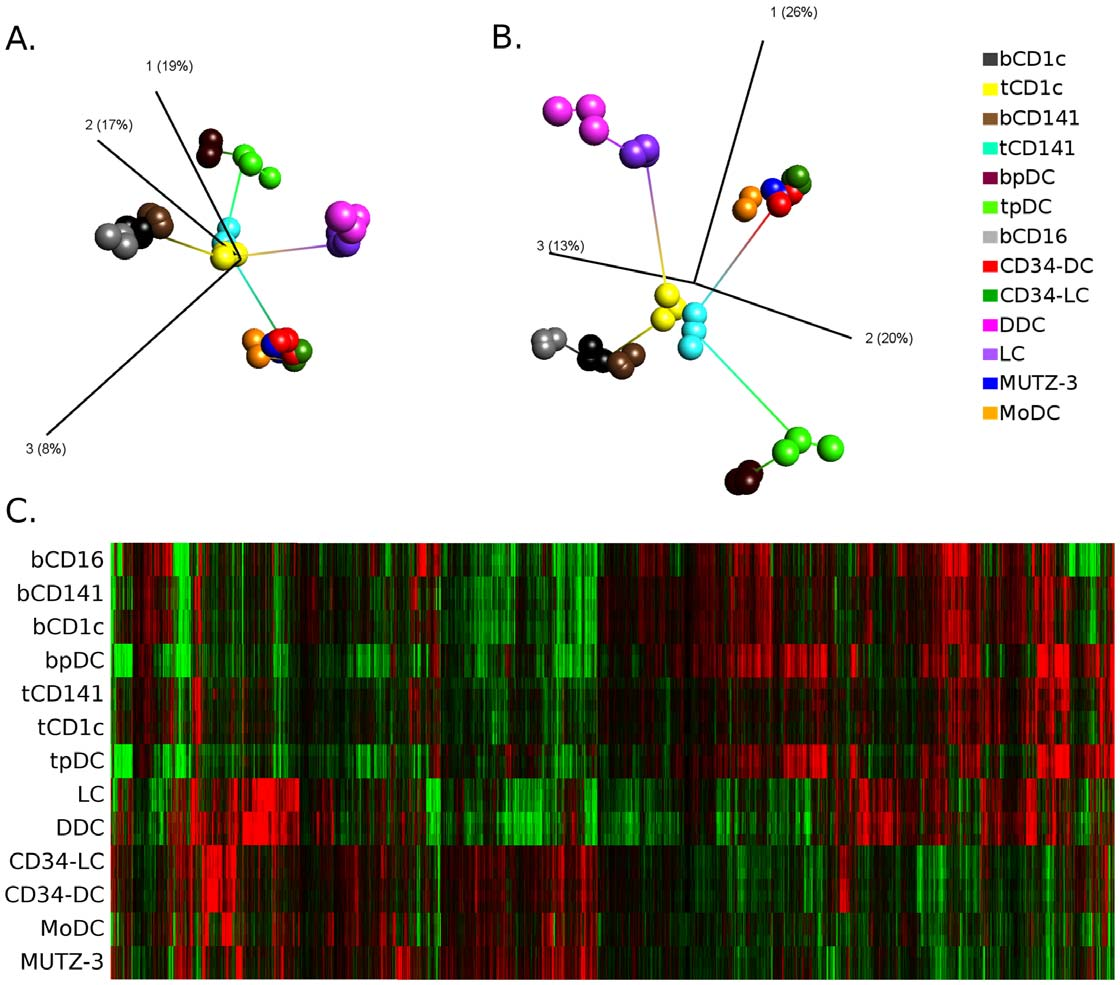
Resemblance of *in vitro* DC models with skin, tonsillar and peripheral blood DC populations. Resemblance demonstrated by principal component analysis (PCA) of expressed transcripts (A. 51,191) and differentially expressed transcripts (B, 18,590) identified by ANOVA p<10^−6^ (corresponding to p<0,05 after Bonferroni correction). Replicate similarities visualized using *k*-Nearest Neighbors (*k*-NN) algorithm (*k* = 2 in this case) and relationships between cell types demonstrated by minimal spanning tree analysis (lines connecting the different populations). Axes (marked 1, 2 and 3) correspond to the three main components in the PCA analysis and numbers in brackets correspond to percentage of total variation contained within each component. C) Heatmap visualizing gene expression profiles of differentially expressed genes (18,590) upon hierarchical clustering with complete linkage and Euclidean measure. Colors represent high (red) and low (green) normalized intensity.

In total, 51,191 genes were expressed by *in vitro* DC models and *ex vivo* DC populations after background noise elimination (Affymetrix control genes excluded). The PCA analysis performed on these transcripts showed excellent replicate resemblance (Figure 1a). *In vitro* DC models formed a separate cluster and thus, based on the entire set of expressed transcripts, individual model DCs showed extensive similarity to each other. Nevertheless, minimal spanning tree analysis (lines connecting samples - length corresponding to similarity) demonstrated that gene expression profiles of *in vitro* differentiated DC models were most closely related to primary mDCs isolated from tonsillar tissue (Figure 1a). In addition to analysis based on all expressed transcripts, ANOVA analysis was performed in order to identify differentially expressed transcripts among DC samples. Using all samples and a p-value cutoff of <10^−6^ identified 18,590 transcripts. Again, PCA analysis demonstrated excellent replicate similarity and *in vitro* DCs connected to tonsillar mDCs upon minimal spanning tree analysis (Figure 1b).

To obtain a graphical outline of *in vitro* and *ex vivo* DCs' transcriptional profiles, a heatmap was created, based on hierarchical clustering performed on differentially expressed genes, using Euclidean distance and complete linkage (Figure 1c). CD34-DCs and CD34-LCs shared many expressed genes among themselves, in line with these DC models being derived from the same precursor cell. In contrast, when compared to MoDCs and MUTZ-3 DCs larger deviations were apparent. Also, transcripts expressed by *in vitro* DCs showed different patterns across primary DC populations. Regarding primary DCs, transcriptional profiles of mDC populations from the same source or tissue (blood, tonsil or skin, respectively) showed extensive similarities, suggesting large micro-environmental influences on mDCs. In contrast, pDCs appeared more rigid to environmental influence, as the transcriptomes of pDC from blood and tonsils displayed large resemblance.

### Heterogeneous gene expression profiles among *in vitro* DC models, as well as among *ex vivo* DC populations

**Figure 2:**
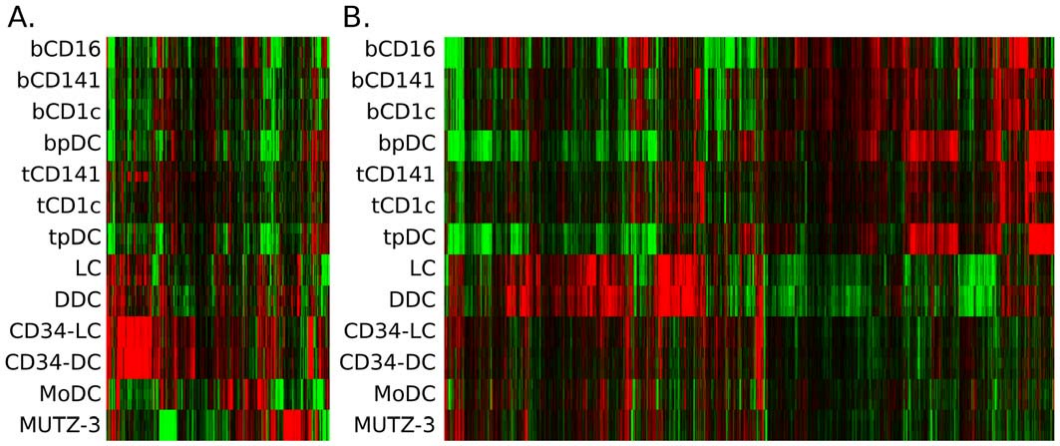
Heatmaps visualizing gene expression profiles of *in vitro* DC models and *ex vivo* DC populations. Hierarchical clustering on differentially expressed transcripts (ANOVA p<10^−6^, corresponding to p<0,05 upon Bonferroni correction) among *in vitro* DCs (A. 892 transcripts) and *ex vivo* DCs (B. 9,055 transcripts), using complete linkage and Euclidean measure. Colors represent high (red) and low (green) normalized intensity, respectively.

To appreciate differences between distinct *in vitro* DC models and to understand the degree to which model DCs reflect differences among *ex vivo* DC populations, transcripts differentially expressed among *in vitro*, as well as transcripts differentially expressed among *ex vivo* populations, were identified. ANOVA analysis (p<10^−6^) demonstrated that 892 and 9055 transcripts differed among *in vitro* DCs and *ex vivo* DCs, respectively. The higher number of transcripts differing between *ex vivo* DC populations was in line with more samples being compared in that set. Heatmap visualization of transcripts differing among *in vitro* DCs demonstrated variability across *ex vivo* DC populations (Figure 2a). Moreover, clustering on transcripts differing among *ex vivo* DC populations (9055) showed discrepant expression levels across *in vitro* DC samples, although regions of overall low expression by *in vitro* DCs were also identified (Figure 2b). Importantly, 435 of the 892 transcripts differing among *in vitro* DC models were additionally found to differ among *ex vivo* DCs (data not shown). Taken together, although the overall gene expression profiles of *in vitro* DCs were more similar to other model DCs than to primary DCs (Figure 1a and b), there may be important differences that can affect specific functionality. Consequently, individual model DCs could differentially reflect specific features of primary DCs. To understand the applicability of distinct *in vitro* DC models, further insight into expression of immune-related transcripts by *in vitro* DC models as well as *ex vivo* DC subsets is required.

### Overall relationship of *in vitro* DC models

**Figure 3:**
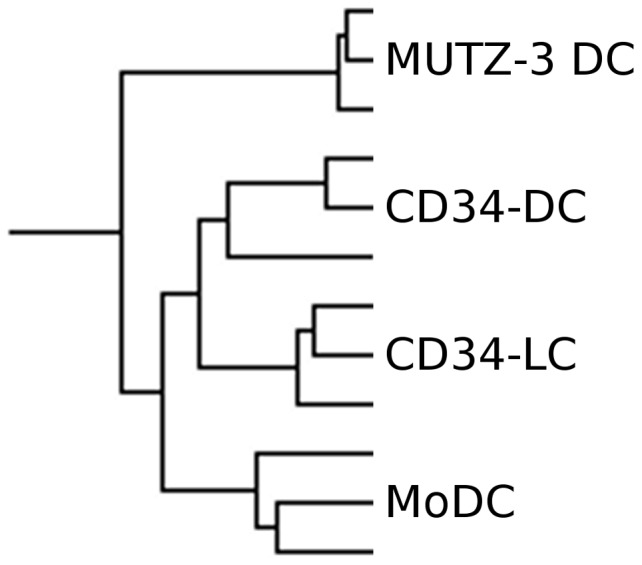
Hierarchical clustering of *in vitro* DCs. Clustering using complete linkage algorithm on differentially expressed transcripts (892), identified by ANOVA (p<10^−6^, corresponding to p<0,05 upon Bonferroni correction), demonstrates relationships among *in vitro* DC models.

Hierarchical clustering was performed in order to investigate the relationships between *in vitro* DC subsets and their functional specialization. In line with the PCA analyses, unsupervised hierarchical clustering of genes differentially expressed by *in vitro* DCs (892 genes) demonstrated excellent replicate performance (Figure 3). Additionally, MUTZ-3 DCs formed a separate branch, whereas DCs differentiated from donor-derived cells clustered together.

### Immune-related transcripts expressed by *in vitro* DC models and identification of transcriptional signatures

**Figure 4:**
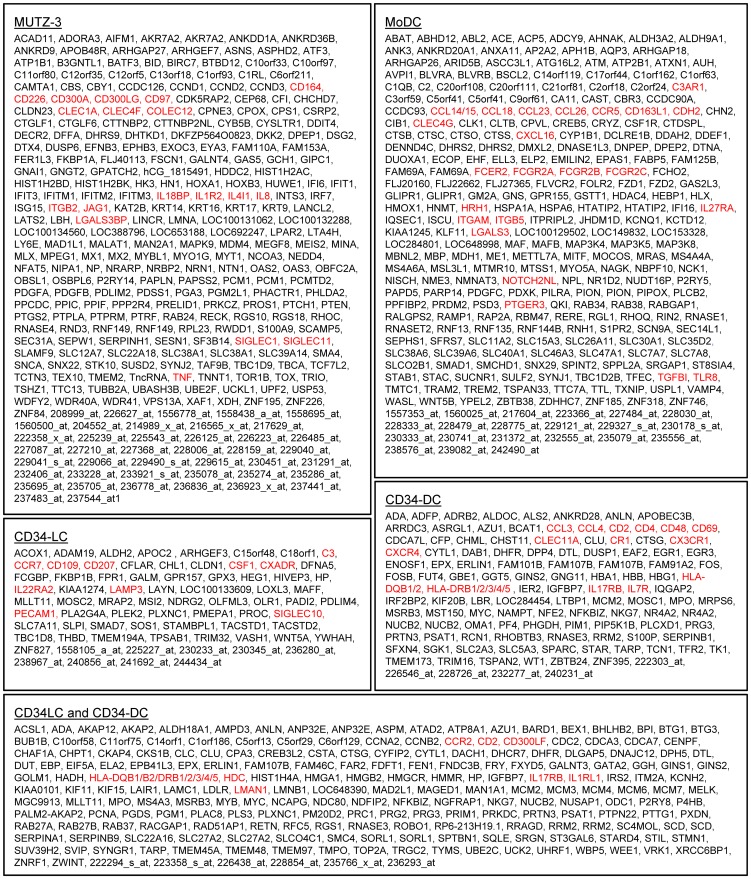
Transcriptional signatures of individual *in vitro* DC subsets. Signatures identified by expression level >200 and differential expression in one model as compared to all other *in vitro* DC models (based on fold difference >2 and statistical significance p<0,05; student's T-test). Comparisons performed on MAS5-normalized data and expression ratio calculated on average of replicates as described in Methods. Selected immununologically associated transcripts are highlighted in red. Transcripts lacking official gene symbols are identified with respective Affymetrix Probe Set ID.

To pinpoint specific features of the distinct *in vitro* DC models as compared to other model DCs, transcriptional signatures were identified for each DC model (Figure 4). Sets of 355 and 341 model-selective transcripts were identified for MUTZ-3 DCs and MoDCs, respectively. In line with CD34-DCs and CD34-LCs being derived from the same precursor cell, less signature genes were identified for these DC models (34 and 89 transcripts, respectively), and when analyzed together 229 transcripts were identified. Identification of distinctive transcriptional signature profiles of *in vitro* DC models is consistent with the heatmap visualizations of differentially expressed transcripts (Figure 2 and 3).

To gain insights into specific DC models' immune functionality and similarity to *ex vivo* DC populations, expression of immunologically associated transcripts were outlined. Important categories of immune-related transcripts were selected and filtered based on expression by one or several DC models. The resulting list of 155 transcripts is presented in Table 2 and corresponding expression levels in *ex vivo* DC populations are additionally shown.

**Table 2:** Expression levels of immunologically important transcripts in model DCs and primary DCs.

		*In vitro* DCs				*Ex vivo* DCs								
						Skin DCs		Tonsillar DCs			Blood DCs			
Probe Set ID	Gene Symbol	MUTZ-3	MoDC	CD34-DC	CD34-LC	DDC	LC	tpDC	tCD1c	tCD141	bpDC	bCD1c	bCD141	bCD16
**Antigen-recognition**														
204438_at	CD206/MRC1	+++	+++	+++	+++	+++	−	−	++	+	−	++	−	−
220428_at	CD207/Langerin	+	−	−	+++	−	+++	−	+++	+++	−	−	−	−
207277_at	CD209/DC-SIGN	+++	+++	+++	++	+	−	−	−	−	−	−	−	−
203799_at	CD302/DCL-1	−	+++	++	++	+++	+++	++	++	++	+++	+++	+++	+++
219761_at	CLEC1A	++	−	−	−	−	−	−	−	−	−	−	−	−
206682_at	CLEC10A/CLECSF14	+++	+++	+++	+++	+++	+	−	+++	+++	+	+++	++	−
211709_s_at	CLEC11A/CLECSF3	+	+	++	+	−	−	−	−	−	−	−	−	−
1552398_a_at	CLEC12A/B/DCAL-2	−	+	++	+	−	−	−	+	−	−	++	+	+++
209732_at	CLEC2B/CLECSF2	++	++	++	+	+++	+++	+++	+++	+++	+++	+++	+++	+++
219947_at	CLEC4A/DCIR	+++	+++	+++	+++	+++	+++	+++	+++	+++	+++	+++	+++	+++
1552552_s_at	CLEC4C/BDCA2	−	−	−	−	−	−	+++	−	+++	+++	−	+	−
1552410_at	CLEC4F	+	−	−	−	−	+	−	+	+	−	−	−	+
1559065_a_at	CLEC4G	+	+++	+	−	−	−	−	−	−	−	−	−	−
219890_at	CLEC5A/MDL1	−	−	−	+	+++	+++	−	−	−	−	−	−	−
221698_s_at	CLEC7A/Dectin-1	+++	+++	+++	+++	+++	+++	−	+++	+++	−	+++	+++	+++
244413_at	CLECL1/DCAL-1	++	++	−	−	−	−	+	+	+	−	−	−	−
221019_s_at	COLEC12	+++	+	+	−	−	−	−	−	−	−	−	−	−
211734_s_at	FCER1A	−	+++	+++	+	+++	+++	++	+++	++	+++	+++	+	−
204232_at	FCER1G	+++	+++	++	++	+++	+	+++	+++	+++	+++	+++	++	+++
206759_at	FCER2/CD23	++	+++	+	−	−	−	−	−	−	−	−	−	−
203561_at	FCGR2A	−	++	+	−	+++	+	−	+	−	−	++	−	+++
210889_s_at	FCGR2B	−	+++	+	−	+++	++	−	+	−	−	+	−	−
211395_x_at	FCGR2C	−	+++	+	−	+++	++	−	+	−	−	+	−	++
218831_s_at	FCGRT	++	+++	+	+	+	++	+	++	++	++	+++	+++	++
37408_at	MRC2	+	+	−	++	−	−	−	−	+	−	−	+	−
224983_at	SCARB2	++	++	+	+	+	+	+++	+	++	+++	++	−	+++
206995_x_at	SCARF1	+	−	−	−	+	+	−	−	−	−	−	−	−
210176_at	TLR1	+	−	−	+	−	−	+	++	++	−	−	−	+
204924_at	TLR2	+	+	++	+	+++	−	−	++	++	−	++	−	+++
224341_x_at	TLR4	−	+	−	−	−	−	−	−	−	−	−	−	+
229560_at	TLR8	−	+++	−	−	−	−	−	+++	+++	−	+	+	+++
**Interleukines and their receptors**														
209827_s_at	IL16	+	−	−	−	+	−	++	++	++	++	++	++	+
206295_at	IL18	−	−	−	+	+	+	−	++	++	−	+	−	−
222868_s_at	IL18BP	+++	+	+	−	−	++	−	+	++	−	+	−	−
39402_at	IL1B	++	−	+	++	+++	+++	+	+++	+++	−	−	−	−
212659_s_at	IL1RN	+	−	−	+	++	−	−	−	−	−	−	−	−
211506_s_at	IL8	+	−	−	−	+++	+	−	−	−	−	−	−	−
207008_at	IL8RB	+	−	−	+	−	−	−	−	−	−	−	−	−
204912_at	IL10RA	+++	+++	+++	+++	+++	+++	+++	+++	+++	+++	+++	+++	+++
209575_at	IL10RB	+	+	+	+	−	−	+	+	+	+	+	−	++
207160_at	IL12A	−	−	−	−	−	−	−	−	−	−	−	−	−
207901_at	IL12B	−	−	−	−	−	−	−	−	−	−	−	−	−
201887_at	IL13RA1	+++	+++	+++	+++	+++	+++	++	+++	+++	++	+++	++	+
205707_at	IL17RA	+	+	+	+	−	−	−	+	+	−	+	−	+
224361_s_at	IL17RB	−	−	++	+	−	−	−	−	−	−	−	−	−
202948_at	IL1R1	++	+++	+++	+++	+++	+++	−	+++	++	−	+	−	−
205403_at	IL1R2	+++	++	++	+++	+++	+++	−	+++	++	−	+++	−	−
205227_at	IL1RAP	+++	+	+	+++	+	++	−	−	−	−	−	−	−
242809_at	IL1RL1	−	−	+	+	−	−	−	−	−	−	−	−	−
237493_at	IL22RA2	−	−	−	+	−	−	−	+++	+++	−	−	−	−
222062_at	IL27RA	+	+++	+	+	+	−	−	++	++	−	++	−	−
206148_at	IL3RA/CD123	−	+	−	−	−	−	+++	−	+	+++	−	−	++
203233_at	IL4R	+	+	+	+	++	+	−	+	+	+	+	−	−
226333_at	IL6R	++	++	++	++	++	++	+	++	++	+	++	++	−
212195_at	IL6ST	++	+++	++	+++	+++	+++	+++	+++	+++	+++	+	+	+
226218_at	IL7R	+	+	+++	++	+++	+	−	+++	++	−	−	−	−
**Signaling molecules**														
209906_at	C3AR1	−	+++	++	+	++	−	−	+	−	−	−	−	+++
203104_at	CSF1R	++	+++	+	+++	+	+++	−	++	++	−	++	−	+++
210340_s_at	CSF2RA	−	+	−	+	+	−	+	++	++	+	−	+	−
205159_at	CSF2RB	+++	+++	+++	+++	+++	+++	+++	+++	+++	+++	+++	+++	+++
1553297_a_at	CSF3R	−	−	+	−	−	−	−	−	−	−	+	−	−
205579_at	HRH1	−	++	−	−	−	−	−	−	−	−	−	−	−
225669_at	IFNAR1	+	+	+	+	−	−	+	+	+	+	−	−	+
204786_s_at	IFNAR2	−	+	−	−	−	−	+	−	+	+	−	−	+
242903_at	IFNGR1	+	+	−	−	+	+	+	++	+	+	+	+	−
201642_at	IFNGR2	+++	++	++	++	++	++	+	++	+	−	+	+	+++
201105_at	LGALS1	+++	+++	+++	+++	+++	+++	+	++	+++	++	+++	+++	+++
208949_s_at	LGALS3	+++	+++	+++	+++	+++	+++	−	+++	+++	−	+++	+++	+++
200923_at	LGALS3BP	+++	−	−	−	−	−	−	−	−	−	−	−	−
208933_s_at	LGALS8	++	++	++	+	−	+	+	+	+	++	++	−	+
206631_at	PTGER2	−	+	+	+	+	−	−	−	−	−	−	−	++
210375_at	PTGER3	−	++	−	+	++	+	−	−	−	−	−	−	−
204897_at	PTGER4	+	++	+++	+++	+++	+++	+++	+++	+++	+	+++	+++	+++
224937_at	PTGFRN	+	+	−	+	−	−	−	−	−	−	−	−	−
1552807_a_at	SIGLEC10	+++	+++	+++	+++	+	+++	−	+++	+++	−	++	+++	+++
207113_s_at	TNF	+	−	−	−	+++	++	−	−	−	−	−	−	−
209295_at	TNFRSF10B	++	++	++	++	+++	+	−	+++	++	−	+++	++	++
227345_at	TNFRSF10D	+	−	+	+	+	−	−	+	−	−	+	−	−
238846_at	TNFRSF11A	−	+	−	+	−	−	−	−	−	−	−	+	−
209354_at	TNFRSF14	+	+	+	+	+	+	+	++	+	+	+	+	++
207643_s_at	TNFRSF1A	+	++	+	+	+	+	−	+	+	−	−	−	−
203508_at	TNFRSF1B	−	+	+	−	+++	++	+	++	++	+	+++	+	+++
209500_x_at	TNFSF12/13	+	++	++	++	−	+	++	++	++	++	+++	+	+++
210314_x_at	TNFSF13	+	++	++	++	−	+	++	++	++	++	+++	+	+++
223501_at	TNFSF13B	++	+++	+++	+	+++	−	+++	+++	+++	+++	+++	+++	+++
**CD antigens**														
205055_at	CD103	+	++	++	++	+	+	+++	++	+++	+++	+++	++	+
226545_at	CD109	+	+	+	++	++	+++	−	−	−	−	−	−	−
201743_at	CD14	−	+	+	−	+	−	−	−	−	−	−	−	−
205831_at	CD2	−	−	++	+	−	−	−	+	+	−	++	−	−
207315_at	CD226	++	+	−	+	−	−	−	−	−	−	−	−	−
266_s_at	CD24	+++	−	+++	++	−	−	−	−	−	−	−	−	−
224859_at	CD276	+	+	−	+	−	−	−	−	−	−	−	−	−
211945_s_at	CD29	+++	+++	+++	+++	+++	+++	+++	+++	+++	+++	+++	+++	+++
209933_s_at	CD300A	+++	−	+	−	+	+	+++	+++	+++	++	+++	+	+++
228766_at	CD36	+++	+++	++	+++	+	−	+++	+	+	+++	++	−	−
203547_at	CD4	−	−	+	−	+	+	+++	+++	+++	+++	++	+	+
212063_at	CD44	+++	+++	+++	+++	+++	+++	+++	+++	+++	+++	+++	+++	+++
208783_s_at	CD46	++	+++	+++	+++	+++	+++	+++	+++	++	+++	+++	+++	+++
226016_at	CD47	++	++	++	++	+	++	+++	++	++	+++	++	++	+++
204118_at	CD48	−	−	+++	+	++	−	++	+++	+++	+++	+++	+++	+++
202351_at	CD51	+++	+++	++	+++	+++	++	−	+	+	−	−	−	−
34210_at	CD52	+	+++	+++	++	+++	+++	+	+++	++	++	+++	++	+++
203416_at	CD53	+++	+++	+++	+++	+++	+++	+++	+++	+++	+++	+++	+++	+++
201925_s_at	CD55	+	−	++	+	+++	+++	+++	++	++	+	+++	+	++
211744_s_at	CD58	+	++	+	++	+++	+++	−	++	++	−	++	+	++
212463_at	CD59	+++	+++	++	++	+	++	−	−	+	−	+	++	−
200663_at	CD63	+++	+++	+++	+++	+++	+++	++	+++	+++	+++	+++	+++	+++
209795_at	CD69	+++	−	+++	++	+++	+++	++	+++	++	+	+	−	−
208691_at	CD71	+++	+++	+++	+++	+++	+++	+++	+++	+++	+++	+++	+++	+++
209619_at	CD74	+++	+++	+++	+++	+++	+++	+++	+++	+++	+++	+++	+++	+++
202105_at	CD79A	++	+	++	++	+	++	+	++	++	++	+++	+++	++
205988_at	CD84	−	+	+	+	+	−	−	+	+	−	−	−	−
201005_at	CD9	−	++	−	++	+++	+++	−	+	−	+	−	−	−
202878_s_at	CD93	++	−	++	++	++	−	+	+++	+++	−	+++	+++	+
**Chemokines and receptors**														
206407_s_at	CCL13	−	+++	+++	++	−	−	−	−	−	−	−	−	−
205392_s_at	CCL14/15	−	++	−	−	−	−	−	−	−	−	−	−	−
207900_at	CCL17	−	−	−	+	−	−	−	−	−	−	−	−	−
209924_at	CCL18	−	+++	+++	−	−	−	−	−	−	−	−	−	−
207861_at	CCL22	−	++	+	+++	+++	+++	−	+++	+	−	−	−	−
210548_at	CCL23	−	+++	−	−	−	−	−	−	−	−	−	−	−
223710_at	CCL26	−	+++	−	−	−	−	−	−	−	−	−	−	−
205114_s_at	CCL3	−	−	+	−	+++	−	+	+++	++	−	−	−	−
204103_at	CCL4	−	−	+	−	+++	++	−	++	+	−	−	−	−
214038_at	CCL8	−	−	+	−	−	−	−	−	−	−	−	−	−
205098_at	CCR1	+++	+++	+++	+++	−	−	−	+	−	−	−	−	−
206978_at	CCR2	+	−	+++	+++	−	−	+++	++	+	+++	+	−	−
206991_s_at	CCR5	−	+++	+	+	+++	−	+++	+++	++	+	+	−	−
206337_at	CCR7	−	−	−	+	+++	−	−	+++	++	−	−	−	−
219161_s_at	CKLF	+++	++	++	+++	++	+++	+	+++	+++	++	+++	+++	++
205898_at	CX3CR1	−	−	++	−	++	+++	++	+++	+++	−	++	−	+++
223454_at	CXCL16	−	++	−	+	+++	+++	−	++	++	−	+	++	+++
217028_at	CXCR4	+++	++	+++	+++	+++	++	+++	+++	+++	+++	+++	+++	+++
1561226_at	XCR1	−	−	−	−	−	−	−	−	++	−	−	++	−
**Maturation/presentation**														
215346_at	CD40	+	+	−	+	+++	+	−	+	+	−	−	−	−
200675_at	CD81	+++	+++	+++	+++	++	++	++	+++	+++	++	++	+++	++
204440_at	CD83	+	+++	+++	+++	+++	+++	+++	+++	+++	+	+++	+++	+++
205685_at	CD86	+	+	+	+	+++	+++	−	++	++	−	++	+	++
206749_at	CD1B	+++	+++	+++	+++	+++	+	−	+	−	−	−	−	−
205987_at	CD1C	+++	+++	+++	+++	+++	+++	−	+++	+++	−	+++	++	−
210325_at	CD1A	+++	+++	+++	+++	+++	+++	−	++	+	−	−	−	−
215784_at	CD1E	+++	+++	++	+++	+++	+++	−	+++	+++	−	++	−	−
205569_at	CD208/LAMP3	−	+	+	+++	+++	+++	−	+++	+++	−	−	−	−
**Adhesion**														
205786_s_at	CD11b	+++	+++	+++	+++	+++	−	−	+	−	−	+	−	+
210184_at	CD11c	−	+	−	+	+	−	−	++	++	−	++	+	+
208654_s_at	CD164	+++	++	++	++	+	++	+++	++	++	+++	++	++	++
206120_at	CD33	++	+++	+	+	−	−	−	+	−	−	+++	+	+
206493_at	CD41	−	−	+	−	−	−	−	−	−	−	−	−	−
213416_at	CD49D	+++	−	++	++	−	−	+++	+++	+++	+++	+++	+++	+++
202910_s_at	CD97	++	−	+	−	++	++	+	++	++	+	++	+	+++
201028_s_at	CD99	+	++	+	+	++	+	+	−	−	++	+	+	−
215485_s_at	ICAM1	+	−	−	+	−	+	−	+	+	−	−	−	−
204949_at	ICAM3	+++	+	+++	+++	+++	+++	++	+++	+++	+++	+++	+++	+++
204563_at	SELL	−	−	+	−	−	−	+++	+++	+++	+++	+++	+++	+
209879_at	SELPLG	+++	++	++	+	+	+	+++	++	+++	+++	+++	+++	+++
44673_at	SIGLEC1	+	−	−	−	−	−	−	−	−	−	−	−	−
1552910_at	SIGLEC11	+	−	−	−	−	−	−	−	−	−	−	−	−
207224_s_at	SIGLEC7	−	−	−	+	−	−	−	+	+	−	+	−	−
**Others**														
202888_s_at	CD13	+++	++	++	+++	+++	−	−	+	++	−	+	+++	+
230966_at	IL4I1	++	−	+	−	+++	+	−	++	++	−	−	−	−
224629_at	LMAN1	++	++	+++	+++	++	++	+++	++	++	+++	++	++	++
200805_at	LMAN2	++	++	++	++	+	+	++	+	+	++	+	+	+
200901_s_at	M6PR	+++	+++	+++	+++	++	++	+++	+++	+++	+++	+++	+++	+++
210004_at	OLR1	−	−	−	+	−	−	−	−	−	−	−	−	−
201819_at	SCARB1	++	+	+	−	−	−	−	−	−	−	−	−	−

Expression levels of 155 transcripts encoding TLRs, CD antigens, lectins, TNF molecules, chemokines, interleukins and receptors, selected based on positive expression in any of the *in vitro* DC models.

*Signal intensity levels: −: <200; + : 200–500; ++: 500–1000; +++: >1000.*

MoDC – monocyte-derived dendritic cell; DDC - Dermal DC; LC - Langerhans Cell; pDC – plasmacytoid dendritic cell; CD34-DC – *In vitro* derived dendritic cell (from CD34^+^ precursor); CD34-LC - *In vitro* derived Langerhans Cell (from CD34^+^ precursor).

PCA analysis on the 155 identified transcripts showed that *in vitro* DCs clustered together and were most closely related to tonsillar mDCs, (data not shown), in line with PCA analyses on expressed and differentially expressed genes (Figure 1a and b). This supports the previous suggestion that developing model DC equivalents of specific *ex vivo* DC populations may require additional signals, such as influence of tissues-specific micro-environmental factors. Nevertheless, differential expression was demonstrated across *in vitro* DC models for several immune-related transcripts, and similarly among *ex vivo* DCs (Table 2). Importantly, the identified model DC signatures included several immunologically associated molecules (highlighted in red in Figure 4). Thus, individual DC models can be expected to have different abilities to respond to specific stimuli, as well as to stimulate immune responses downstream of DCs. Consequently, suitability of DC models in different applications should be judged on a case-by-case basis and be decided by the expression of transcripts relevant to the research question. Also, attempts to develop model DCs with enhanced resemblance to specific primary DC populations, by exposing them to tissue-specific factors, may have model-specific outcomes. Of note, MUTZ-3 DCs, which have the great advantage of not being dependent on donor material, express numerous immune-related transcripts (Table 2), supporting their suitability in immune applications.

### Applicability of individual DC models based on expression of immune-related transcripts and signatures

A large number of immune-related transcripts were found to differ across both *in vitro* DC models and *ex vivo* DC populations. In addition, immunologically associated transcripts were listed within the distinct signatures of *in vitro* DC models. Accordingly, it is likely that individual model DCs mimic functionality of *ex vivo* DC populations differently.

Many transcripts coding for receptors mediating antigen-interactions, such as Fc receptors, TLRs and C-type lectin receptors, were differentially expressed among DC models and primary DC populations. MoDCs and CD34-DCs were shown to express mRNAs encoding Fcγ-receptors (FcgR) (Table 2 and Figure 4). Moderate to high levels of *FCGR2A*, *FCGR2B* and *FCGR2C* were demonstrated in MoDCs, reflecting levels in primary skin-DCs, whereas CD34-DCs showed lower levels in similarity to primary CD1c^+^ tonsillar and blood DCs (Table 2). Fcγ receptors regulate ongoing immune responses via IgG-mediated antigen uptake [36], and whereas *FCGR2A* and *FCGR2C* encode receptors inducing activating signals, *FCGR2B* code for an inhibitory receptor. Levels of *Toll like receptor (TLR) 1*, *TLR2*, *TLR4* and *TLR8* differed across *in vitro* DCs as well as across *ex vivo* DC populations. *TLR8* was a MoDC signature transcript (Figure 4) and among primary DCs, *TLR8* was detected in tonsillar and blood mDCs (Table 2), suggesting a similar ability to respond to single-stranded RNA [37]. In line with Langerin/CD207 expression being a hallmark of LC differentiation [38], *CD207* mRNA was detected predominantly in CD34-LC (Figure 4) and in primary LCs (Table 2), but was additionally expressed by tonsillar mDCs. Based on recent data, this implies that these DCs are capable of CD207-mediated recognition of fungi [39] and measles virus [40]. *C-type lectin domain family (CLEC) 4, member F* was expressed by primary LCs, tonsillar mDCs and CD16^+^ blood DCs; however the function of the encoded receptors is currently not known. Among model DCs, *CLEC4F* was shown to be uniquely expressed by MUTZ-3 DCs, suggesting these cells to be an appropriate *in vitro* DCs to employ to investigate the role of the receptor.

Transcripts encoding interleukins and their receptors showed different levels and these molecules influence, amongst other things, DCs' stimulation of T-cell responses. For example, the highest levels of *Interleukin (IL) 27 receptor alpha (RA)* were shown in tonsillar mDCs as well as CD1c^+^ blood DCs (Table 2). Regarding *in vitro* DCs, *IL27RA* was listed in the MoDCs transcriptional signature (Figure 4). DC binding of IL-27 is shown to inhibit Th1 responses [41] and such influences appear to be primarily apparent in these primary DCs and MoDCs. Furthermore, CD34-DCs expressed significantly higher levels of *IL7R* than other DC models (Figure 4), and among primary DCs, the highest levels were found in DDCs and tonsillar CD1c^+^ DC (Table 2). IL-7, produced by e.g. stromal cells and keratinocytes [42], [43], is shown to down-regulate DC expression of major histocompatibility complex class II and to lead to diminished CD4^+^ T cell proliferation in mice [44]. However, IL-7 has also been shown to be necessary for efficient interactions between T cells with DCs [45]. Thus, culturing DCs in the presence of IL-7-producing cells may either enhance or limit the induction of T-cell responses by CD34-DCs.

Transcripts coding for many other signalling receptors that influence DC function were differentially expressed by *in vitro* DCs and some were additionally identified as signature transcripts. Of these, several also differed across *ex vivo* DC samples. For example, *prostaglandin E receptor 3 (PTGER3)* was expressed at moderate levels by MoDCs (Figure 4 and Table 2) and similar levels were demonstrated in primary DDCs, whereas CD34-DCs and primary LCs expressed low levels (Table 2). Signalling via the prostaglandin 3 receptor may be involved in the expansion of Th17 cells through stimulation of IL-23 production by DCs [46], [47], thus a likely feature of these DC models and primary DC populations. Moreover, *in vitro* DC models were shown to express different levels of transcripts coding for chemokines and their receptors (Table 2 and Figure 4). Consequently, to mimic migratory patterns of primary DC subpopulations, and their capacity to interact with other cells, chemokine molecules involved should be identified and the mRNA expression levels presented herein will aid in the selection of the appropriate model DC to resemble distinct aspects of a specific *ex vivo* DC population.

The list of immune-related transcripts shown to be expressed by at least one *in vitro* DC model additionally demonstrated a set of genes that were expressed by the entire set of DC types investigated. These include *C-type lectin domain family (CLEC) member 2b* and *CLEC4a/DCIR*, *Fc epsilon RI gamma-chain* (*FCER1G*), *Fc fragment of IgG receptor transporter* (*FCGRT*), *IL10RA*, *IL13RA1*, *IL6ST*, *colony-stimulating factor beta-chain (CSF2RB)*, *Galectin 1 (LGALS1)*, *prostaglandin E receptor 4 (PTGER4)*, *tumor necrosis factor receptor superfamily, member 1A* (*TNFRSF1A*), *CD103*, *CD29*, *CD44*, *CD46*, *CD47*, *CD52*, *CD53*, *CD63*, *CD71*, *CD74*, *CD79A*, *CD81*, *CD83*, *CD164*, *chemokine-like factor (CKLF)*, *chemokine (C-X-C motif) receptor 4 (CXCR4)*, *intercellular adhesion molecule 3 (ICAM3)*, *selectin P ligand (SELPLG)*, lectin mannose-binding (*LMAN*) *1* and *LMAN2*, and *mannose-6-phosphate receptor (M6PR)*. Thus, functional assays dependent on these specific genes may be based on any of the *in vitro* DC models. For example, activation of DCIR can be assumed to have an inhibitory effect in all DC subsets [48], [49]. However, different types of DCs may be differentially affected dependent on responsiveness to the specific stimuli used; i.e. the effect of TLR8-stimulation is inhibited by DCIR triggering in mDCs, whereas TLR9-stimulation of pDCs is affected. Furthermore, the expression of *PTGER4* by all DC types indicates responsiveness by all models and primary DCs to prostaglandin E2, which is shown to promote induction of Th2 responses by DCs [50], [51]. Also, *CD81* was expressed by the entire set of DC types investigated, and as CD81 has been shown to be involved in DC migration [52], each DC model investigated can be predicted to have appropriate CD81-mediated motility. CD83 is a maturation marker that is pre-formed in immature DCs [53], and the expression of *CD83* by all DC types investigated suggests that each DC model shares the capacity of primary DCs to express surface CD83 upon maturation and thereby enhance T-cell stimulation [54].

Overall, the presented overview indicates that differences among model DCs can be expected e.g. in studies dependent on antigen-receptor expression, responsiveness to certain stimuli, ability to activate T-cells and ability to migrate towards specific chemokines. Selection of a DC model for use in co-cultures will be difficult as conditions are more complex compared to single cell cultures, and to a large extent undefined. Furthermore, although the transcriptional profiles of DC samples demonstrated differential expression of many soluble mediators, such as *tumor necrosis factor* (*TNF)*, *IL8*, *IL16*, and *IL6*, specific triggers may be required to induce their secretion. Therefore, functional outcomes related to these transcripts are difficult to predict. Nevertheless, data presented herein gives important indications on functional distinctions among *in vitro* DC models and also their resemblance to specific *ex vivo* DC populations at steady-state conditions. Furthermore, the results can guide investigations of mechanisms underlying functional behavior.

### Development of *in vitro* test assay for prediction of sensitization

The pivotal role of DCs in allergic responses and the cell number required suggests that model DCs are appropriate as cellular basis for alternative *in vitro* test assays for prediction of sensitization. These should ideally mimic primary DC responses. However, the transcriptional profiling analysis showed great diversity among primary DC populations and their importance for sensitization is not fully clarified. Based on frequency alterations in peripheral blood and/or airway tissues upon allergen challenge, CD1c^+^ and pDCs have been argued to be important antigen-presenting cells in allergic responses [55]–[57]. Moreover, increased levels of CD141^+^ DCs have been detected in peripheral blood of allergic as compared to non-allergic controls, and CD141 is upregulated on MoDCs and pDCs upon allergen challenge, supporting a role of this receptor [55], [58], [59]. CD1c^+^ DCs as well as skin LCs and DDCs may be implicated in the perpetuation of allergic responses based on expression of the high affinity IgE receptor (FcεRI), which augments Th2 responses [58], [60], [61]. Expression of *Fc epsilon RI alpha-chain* (*FCERIA*) was detected in all models except MUTZ-3 DCs, and high levels were demonstrated by MoDCs and CD34-DCs. However, involvement of FcεRI during sensitization is unlikelyas no antigen-specific IgEs are present in this phase. In addition to the role of specific DC populations, efforts have been made to identify central processes involved in sensitization. For example, DCs have been shown to promote Th2 responses under the influence of thymic stromal lymphopoietin (TSLP) and prostaglandin E2 (PGE2) [50], [62] and the effects are mediated via the TSLP receptor and prostaglandin E receptor 4 (PTGER4), respectively [51]. Taken together, knowledge on the role of distinct DC populations in the sensitization process *in vivo* is incomplete and central aspects to be mimicked by model DCs remain to be fully defined. However, *PTGER4*, was shown to be expressed by each *in vitro* DC model analysed (Table 2), thus indicating expression of molecules important in sensitization. Indeed, MoDCs, CD34-derived DCs and MUTZ-3 cells have all shown potential to discriminate skin sensitizers from non-sensitizers [11], [63]–[65]. For example, predictive assays based on CD34-derived DCs [63] as well as MUTZ-3 cells [11] have been developed with estimated high accuracies (both assessed by cross-validation). Thus, DC models show great promise as cell bases in allergenicity test assays and future studies will demonstrate whether the *in vitro* assays can be extended to determine respiratory sensitization by chemicals and proteins.

### Applicability of model DCs in treatment of immune disorders based on transcriptional profiles

Upon employment of *in vitro*-derived DCs for treatment of diseases such as cancer, autoimmune diseases and HIV-AIDS, specific features are required rather than simply primary DC mimicry. For example, antigen loading and the ability to obtain stable mature or immature model DCs are important. Thus far, MoDC is the most commonly employed DC model in this context. However, MUTZ-3 DCs have the advantage of being donor-independent and their transcriptional profile can be used for focused development of modulation strategies.

Induction of antigen-specific responses using DC-based immunotherapy is dependent on antigen internalization, and this process is mediated e.g. via Fc receptors and C-type lectin receptors. As previously mentioned, MoDCs were shown to express high levels of several IgG receptors (Table 2) and some were also identified as signature transcripts (Figure 4), thus indicating superior IgG-mediated antigen internalization by this DC model. However, activating signals mediated by FCG2A and FCGR2C may be counteracted as the inhibitory *FCGR2B* was also expressed by MoDCs. Nevertheless, antigen loading by targeting Fcγ-receptors on DCs has been shown to increase the efficacy of anti-tumour responses in mice [66]. Additionally, based on the transcriptional profiles, antigen loading via C-type lectin receptors may be explored (Table 2 and Figure 4). For example, targeting CLEC4G on MoDCs, CLEC4F on MUTZ-3 DCs and CLEC11A on CD34-DCs could be useful strategies to mediate antigen uptake based on the transcriptional signatures identified. However, among these receptors, only CLEC4G has thus far been shown to mediate internalization to our knowledge [67]. Importantly, uptake via C-type lectin receptors have been shown to trigger immunity or tolerance depending on the specific receptor [68]–[71] and targeting different C-type lectin receptors may thus be useful in treatment of both insufficient and detrimental immune responses.

Adequate maturation is of vital importance to evoke immunity and therefore important in model DC based treatment of e.g. cancer and infectious diseases. This is a delicate matter as immature DCs induce tolerance [24], [25] and excessive stimulation can lead to exhaustion [72]. The standard maturation cocktail in MoDC-based immunotherapy of cancer includes IL-1β, IL-6, TNF-α and PGE2 [73]. Similarly, this is the most commonly used DC model and maturation stimuli in treatment of HIV-1, although only a limited number of clinical trials have thus far been undertaken [20]. In line with the maturation strategy, MoDCs express mRNAs which encode receptors responsive to these mediators (Table 2). Interestingly, TLR7/8-induced maturation has been suggested to improve efficacy of cancer therapy as it promotes increased secretion of IL-12 [74]. *TLR8* was identified as a MoDC signature transcript (Figure 4), suggesting this strategy to be primarily suited for MoDCs. Based on the transcriptional profiles in Table 2, TLR2 and/or TLR4 ligands may additionally be explored for induction of MoDC maturation. Generally, *in vitro* DC models showed distinct transcriptional profiles with regards to receptors that induce maturation, thus suggesting that successful maturation strategies are model-specific.

In order to inhibit or limit detrimental immune responses using DC-based therapy, e.g. in treatment of inflammatory diseases, immature status is desirable [24], [25]. However, mediators present at the inflammatory site *in vivo* may lead to maturation of immature model DCs and therefore, a more rigid tolerogenic phenotype is required. Various agents, such as IL-10, TGF-β and macrophages colony stimulator factor, have been assessed for development of tolerogenic DCs in treatment of autoimmunity, but no consensus has thus far been reached [22]. The transcriptional profiles can be used to improve DC modulation and/or to choose the appropriate *in vitro* DC model to induce tolerogenic immune responses. For example, stimulation of CD47 as well as IL-7 and IL-27 receptors, expressed at mRNA levels by *in vitro* DC models (Table 2), have been shown to inhibit Th1 responses by DCs [41], [44], [75], and treating model DCs with corresponding ligands may thus be effective for inhibiting Th1-mediated inflammatory responses. Taken together, the unique transcriptional signatures and expression of immune-related transcripts outlined for individual *in vitro* DC models can direct future advances in DC-based immunotherapy and thereby improve clinical efficacy.

### Summary

By using global transcriptional analysis we have gained insights into the relationship and specific characteristics of MUTZ-3 DCs, MoDCs, CD34-LCs and CD34-DCs, in comparison to each other as well as to nine primary DC populations from skin, tonsil and blood. PCA analysis demonstrated that each *in vitro* DC model was most closely related to tonsillar mDCs among the *ex vivo* DCs populations and induction of transcriptional programs reflecting other tissue-specific primary DC subsets may require micro-environmental factors. Although model DCs were more similar to each other than to primary DCs, expression levels of many of the 155 immune-related transcripts shown to be expressed by *in vitro* DC models, differed across DC models as well as *ex vivo* DC populations. Additionally, model-specific signatures were identified for each *in vitro* DC and these contained several immunologically associated genes. Thus, model DCs are likely to have distinct immune functionalities and thus different applicability in test assays, in clinical settings and as research tools in order to understand DC biology.

## Supporting Information

Figure S1 **Activation status and gating strategy for sorting of MUTZ-3 DCs.** After differentiation *in vitro*, an immature phenotype was confirmed in FACS analysis (A). For sorting, cells were gated on live cells in forward and side scatter and further gated for HLA-DR^+^CD1a^+^ cells (B).
(TIF)

Figure S2 **MoDC phenotype.** MoDCs showed appropriate immature phenotypes with expression of HLA-DR and CD1a, but lack of CD14 and very few cells positive for CD86.
(TIF)

Figure S3 **CD34-LC and CD34-DC phenotypes.** CD34-DCs and CD34-LCs showed positivity for CD1a, whereas expression of CD14 was very low. A considerable portion of the CD34-LC population, but not the CD34-DC population, was positive for CD207/Langerin. Both DC models showed immature phenotypes with very low expression of CD86 and CD83.
(TIF)
